# Undoing resilience: immigrant status and poor health following incarceration

**DOI:** 10.1186/s40352-021-00129-7

**Published:** 2021-02-05

**Authors:** Julie L. Kuper, Jillian J. Turanovic

**Affiliations:** College of Criminology and Criminal Justice, Florida State University, 112 S. Copeland Street, Tallahassee, FL 32306 USA

**Keywords:** Foreign-born, Immigrants, Immigrant paradox, Incarceration, Poor health, Self-rated health

## Abstract

**Background:**

In the United States, foreign-born persons often have better health outcomes than their native-born peers, despite exposure to adversity. Nevertheless, it is unclear whether this pattern extends to the consequences of life events, such as incarceration, that separate immigrants from their supportive networks and increase exposure to adversity. Accordingly, using four waves of data from the National Longitudinal Study of Adolescent to Adult Health, hierarchical generalized linear models were used to examine within-individual changes in self-rated health following first incarceration (*N* = 31,202 person-waves).

**Results:**

The results showed that incarceration was associated with modest health declines that were similar in magnitude for immigrant and native-born persons. Supplemental analyses revealed that these effects did not vary by immigrant race or ethnicity, or by age at immigration. The only exception was for immigrants from low- and middle-income countries, who were marginally less likely to experience health declines following incarceration.

**Conclusions:**

In general, incarceration appears to be similarly health damaging for immigrants and non-immigrants. These findings raise important questions about how incarceration is linked to health declines for foreign- and native-born populations and emphasize the importance of access to healthcare for individuals released from correctional facilities. More research is needed, however, to further examine the cumulative impacts of incarceration on immigrants’ health across the life course, and to assess a broader spectrum of health outcomes.

## Introduction

Growing literature illustrates that immigrants to the United States fare better when confronting adversity than native-born individuals. Research routinely shows that foreign-born persons are better able to overcome trauma, disadvantage, and other life stressors, and that they generally have better health outcomes than U.S.-born individuals (DeJonckheere, Vaughn, & Jacquez, 2017; Espinosa et al., 2018; Marks, Ejesi, & Coll, 2014). This pattern is referred to as the “immigrant paradox.” Although there are several explanations for the paradox, one contributing factor is that foreign-born persons have enhanced cultural capital and strong family ties to draw from in times of hardship (Fergus & Zimmerman, 2005; Motti-Stefanidi, 2018). Immigrant communities are often close-knit, allowing foreign-born persons to acquire social support and to cope with adversities in ways that do not compromise their health (Germán, Gonzales, & Dumka, 2009; Goodman, Vesely, Letiecq, & Cleaveland, 2017).

One unique threat to immigrant resilience, however, is incarceration. Unlike many other life stressors, incarceration knifes off individuals from the communities and families that serve to protect them against poor health outcomes (Cochran & Mears, 2013; Wildeman & Wang, 2017). Incarceration can be particularly health damaging in that it exposes individuals to conditions of confinement where they have little autonomy. Furthermore, because American correctional facilities are often overcrowded, the risk of being exposed to disease and violence is high, and daily life can be tremendously stressful (Massoglia, 2008; Massoglia & Pridemore, 2015). Incarceration can also serve as a turning point in the life course that sets in motion a trajectory of accumulating disadvantages that worsen health (Baćak, Andersen, & Schnittker, 2019). Nevertheless, even though we know that incarceration can be harmful to one’s health, it is unclear whether the immigrant paradox would extend to this context. Instead, it is possible that the negative impacts of incarceration may effectively “undo” immigrants’ resilience, precisely because incarceration is intended to separate people—both immigrants and non-immigrants alike—from the structural and social sources of support that may contribute to their resilience.

Accordingly, the purpose of this study is to assess whether the association between incarceration and poor health is similar for foreign- and native-born persons. Using four waves of data from the National Longitudinal Study of Adolescent to Adult Health, we focus on within-individual changes in respondents’ self-rated health following their first incarceration. Although our main focus is on health declines for immigrants and native-born persons after incarceration, we also compare findings across different groups of immigrants (Black and Hispanic immigrants versus those of other races, and those born in low-middle income versus high-income countries). Our findings call into question the generalizability of the immigrant paradox and draw attention to the health consequences of incarceration in America.

## Background

### The health consequences of incarceration

For the approximately 10 million individuals released from U.S. prisons and jails each year, health problems are a common concern (Bronson & Carson, 2019; Dumont, Brockmann, Dickman, Alexander, & Rich, 2012; Zeng, 2019). Rates of mortality, infectious disease, and cardiovascular problems are higher among individuals who have been previously incarcerated (Pew Charitable Trusts, 2017), and health declines can occur both during confinement and after release (Schnittker & John, 2007). These negative health outcomes can be the result of exposure to stress and disease in correctional settings, to the limited access to adequate healthcare that incarcerated individuals face upon release from institutions (Semenza & Link, 2019), and to the proliferation of stigma and stressors over the life course following incarceration (Massoglia, 2008; Schnittker, 2014).

Indeed, the culture within many American correctional facilities encourages aggression to gain status and respect, where problems are solved using physical force (De Viggiani, 2006; Michalski, 2017). Risks of victimization and exposure to violence are thus elevated during incarceration (Wooldredge, 2020). To make matters worse, the stress induced by the prison environment compromises immunity, making incarcerated persons increasingly vulnerable to contracting infectious diseases (Wildeman & Wang, 2017). Some research notes, for example, that more than 80% of incarcerated individuals have some form of communicable health ailment or substance abuse problem that heightens the likelihood of disease transmission (Pew Charitable Trusts, 2017). Further, chronic conditions may be aggravated behind bars due to low-quality food, poor environmental conditions, and limited resources for medical treatment (Novisky, 2018). Rates of hypertension, asthma, mental health disorders, HIV, and vitamin D deficiency are also higher among those in confinement, and the worsening of these chronic conditions while incarcerated negatively affects lifetime health prospects (Fazel & Baillargeon, 2011).

For many incarcerated persons, untreated health concerns are exacerbated upon reentry to the community (Brinkley-Rubinstein, 2013; Semenza & Link, 2019). Individuals exiting correctional facilities often do not receive medical follow-ups, they generally lack adequate provisions for continued medications, and they often lack a primary care physician (Wildeman & Wang, 2017). In addition, formerly incarcerated persons are subjected to social stigma and often barred from using social service programming that might otherwise help them obtain gainful employment and acquire medical insurance (Pager, 2003; Schnittker, 2014; Wakefield & Uggen, 2010). Medical insurance offers preventative care and assists in the early identification of future health problems, yet recent estimates suggest that 80% of released inmates are uninsured (Pew Charitable Trusts, 2017). The negative health consequences of incarceration can therefore compound and proliferate over the life course, constituting a cyclical process of stress, increased disease susceptibility, blocked resource access, and deteriorating health (Semenza & Link, 2019). In the most severe cases, health challenges ignited by incarceration result in increased rates of mortality as individuals reenter society (Rosen, Schoenbach, & Wohl, 2008).

Yet despite the body of research linking incarceration to negative health consequences, not everyone who experiences incarceration is equally susceptible to poor health outcomes. Some individuals are more resilient to these health impacts than others. For example, some research finds that individuals who are white or who have a higher social status in incarcerated settings have more control over their health resources and are less likely to experience health declines (Novisky, 2018; Wang & Green, 2010). Other research notes that women may be more at-risk for certain health problems following incarceration, but that they may also be more likely to have health insurance, social support networks, and income assistance to offset this risk (Freudenberg et al., 2005). Thus, certain groups that have greater access to such resources may display signs of positive adaptability after incarceration. Still, it remains unclear whether incarceration has a health damaging impact on immigrants, a group that is generally more resilient to health risks.

### Incarceration, health, and immigrant status

The “immigrant paradox” refers to the consistent finding that, in the U.S., immigrants (the foreign-born) do better than their native-born peers on an array of health indices, despite their increased exposure to adversity (Kennedy, Kidd, McDonald, & Biddle, 2015; Marks et al., 2014; Mendoza, 2009). Conventional wisdom suggests that immigrants should exhibit more health problems given their high poverty rates, low education levels, less access to healthcare, and exposure to troubled neighborhoods (Cardoso & Thompson, 2010; Wright & Rodriguez, 2014; Ybarra, Ha, & Chang, 2017); yet, the opposite turns out to be true. Research in sociology, epidemiology, and public health has found immigrants to have lower rates of mental health problems, substance abuse, and eating disorders than U.S.-born individuals (Bowe, 2017; Ortega, Rosenheck, Alegría, & Desai, 2000; Salas-Wright et al., 2019). Research also shows that these paradoxical effects decline across generations, where first-generation immigrants are less likely to experience obesity, asthma, and poor health than second- or later-generation individuals (Harris, 1999; Nguyen, 2006; Portes & Rumbaut, 2001).

There have been several explanations put forth for the immigrant paradox. These explanations range from self-selection into migration to participation in fewer risky health behaviors (Marks et al., 2014; Wright & Rodriguez, 2014). But there is also evidence to suggest that immigrants possess certain protective factors, or resources, that promote positive adaptation to adversity, such as cultural capital (DeJonckheere et al., 2017; Motti-Stefanidi, 2018). The cultural capital available to immigrants includes a protective ethnic identity, cultural flexibility, and higher rates of family involvement (Perreira, Chapman, & Stein, 2006). With respect to ethnic identity, adherence to heritage cultural values can enhance immigrants’ sense of self, bolster the ability to self-select into positive life circumstances (Espinosa et al., 2018), and inspire ethnic pride and positive self-esteem (Cardoso & Thompson, 2010). Cultural flexibility can be influenced by the multiculturalism of immigrants as they adapt to a new host culture. Research suggests that an ability to speak different languages and to develop social competence is beneficial in a diverse world, supporting mental adaptability, problem-solving skills (Kumi-Yeboah, 2016; Trueba, 2002), and healthy coping techniques in response to adversity (Fergus & Zimmerman, 2005).

Moreover, immigrants may settle in ethnic enclaves where there is a concentration of foreign-born persons. Having access to a network of others who share national origin or a common language allows some immigrants to create strong support networks and to acquire social capital (Goodman et al., 2017). Social networks often promote health, and strong social ties within the community can connect immigrants to healthcare services and reduce the stress and uncertainty of resettlement (Devillanova, 2008; Edge, Newbold, & McKeary, 2014). In addition to their ethnic community, family ties also serve as important sources of resilience and support among foreign-born individuals (Cardoso & Thompson, 2010; Perreira et al., 2006). Values of familism within immigrant communities often emphasize strong attachments to the family unit and ensure that the family continues to be a strong source of support and guidance throughout times of hardship (Germán et al., 2009). For these reasons, immigrants may be able to cope with and overcome adversities in ways that do not compromise their health.

Accordingly, the immigrant paradox would suggest that foreign-born individuals are less likely to suffer the potentially negative health consequences linked to incarceration. The literature on cultural capital, ethnic identity, and familism imply that, during incarceration and upon release, foreign-born persons may have access to many health promoting resources. Family support, for example, can help ease the pains of imprisonment, reduce stress and feelings of isolation, and increase the likelihood of securing stable housing and accessing treatment services post-release (Berg & Huebner, 2011; Wolff & Draine, 2004). It is also possible that immigrants are more resilient to many of the health damaging effects of incarceration given that the conditions in their countries of origin may be similar to, or worse than, those within American correctional facilities. To the extent that immigrants have already been exposed to poor living conditions, inadequate nutrition, social unrest, and violence within their sending countries, it is possible that incarceration represents less of a “shock” to their system, and that its effects on health are more subdued (Norris & Murrell, 1988; Turney, 2017).

### Incarceration as “undoing” immigrant resilience

There is, however, an alternative hypothesis that can be derived from the literature: that incarceration is a life stressor that can undermine immigrants’ resilience. Put differently, it is possible that immigrants are just as likely (or even more likely) than native-born persons to experience health declines following incarceration. As we discussed, incarceration is unlike many life stressors in that it removes individuals from their communities and separates them from their families and sources of social support—the very factors that help immigrants remain healthy in the face of adversity. Further compounding matters is that there are strong anti-immigrant attitudes within American society, and immigrants are subject to hostility and discrimination (Becerra, 2016; Light, Massoglia, & King, 2014). Such hostile attitudes may enhance the stigma of incarceration for foreign-born persons, erode their cultural capital, and lead to poor health outcomes.

Even though immigrants are less likely to engage in crime, to be incarcerated, and to recidivate than native-born citizens (Bersani, 2014; Bersani, Loughran, & Piquero, 2014; Ousey & Kubrin, 2018), there is a growing number of immigrants becoming involved with the criminal justice system due to “crimmigration” policies (Immigration & Customs Enforcement, 2018). Crimmigration is used to describe the ways in which immigration control and criminal justice goals are increasingly intertwined (Eagly, 2017; Light et al., 2014). “Illegality” is defined by government yet experienced in the form of legislation that increases the difficulty of immigrants to become U.S. citizens, restricts federal support for immigrant families, and expands the range of deportable offenses (Becerra, Wagaman, Androff, Messing, & Castillo, 2017; Chavez, 2008). These policies tend to be driven by anti-immigrant sentiment and the false belief that immigrants are inherently dangerous, that they threaten the American economy, or that they seek to take political power away from the dominant majority (Chavez, 2008). Foreign-born persons are thus predisposed to experience discrimination in the form of differential treatment or the denial of opportunities (education, employment, housing). On top of this, the added stigma of incarceration can lead to even more biased or hostile treatment, which can impact health. Discriminatory events have been shown to increase stress and worsen well-being among immigrants (Ayón, Marsiglia, & Bermudez-Parsai, 2010; Becerra, 2016).

Even within immigrant communities, foreign-born persons can be subject to differential treatment and social exclusion due to incarceration. Anti-immigration policies and increased immigration enforcement at the federal, state, and local levels have created fear, anxiety, and confusion within immigrant enclaves—particularly with respect to deportation (Becerra, 2016). Immigrants who fear deportation tend to avoid contact with law enforcement (Reina, Lohman, & Maldonado, 2014) and to distance themselves from those who engage in illegal activities. The friends and family members of incarcerated immigrants may therefore be reluctant to maintain contact during incarceration (visits and phone calls) and may even avoid formerly-incarcerated persons upon their reentry to the community. As a result, incarcerated immigrants may lose their sources of support or social capital and face increased risks of health declines.

### Current focus

Despite the wealth of literature on the immigrant paradox, it remains unclear whether foreign-born individuals are more resilient to the health damaging impacts of incarceration than native-born persons. Incarceration is unlike many life stressors in that it separates immigrants from their families and social capital that help them remain healthy in the face of adversity. Foreign-born persons may also be doubly stigmatized due to their incarceration *and* their immigrant status, which can negatively impact their health. It is unclear whether we will find support for the immigrant paradox in this context, or whether we will find that immigrants and non-immigrants experience similar health declines following incarceration. Accordingly, the primary objective of this study is to determine whether the association between incarceration and poor health varies between immigrants and native-born persons.

## Methods

### Data

The data for this study were drawn from waves 1–4 of the National Longitudinal Study of Adolescent to Adult Health (Add Health)—an ongoing, nationally representative study of individuals enrolled in middle and high school during the 1993–1994 academic year. Add Health was chosen for the current study for three primary reasons: (1) it includes a diverse sample of immigrants and native-born individuals, (2) it contains a sufficient number of individuals who experience incarceration, and (3) its panel-based, longitudinal design allows us to examine within-individual changes in health after incarceration. Waves 1–4 of Add Health span approximately 14 years, and respondents are followed from mid-adolescence through early adulthood.

Add Health began with a sample of 80 high schools and 52 feeder middle and junior high schools, selected through a disproportionately stratified, school-based clustered sampling design (Harris, 2013). The sample was representative of U.S. schools in terms of region, urbanicity, school type, school size, and ethnic composition. In the first phase of data collection, a brief questionnaire was administered to all youth enrolled in grades 7–12 in the 132 schools. From the initial in-school survey, a sample of more than 20,000 students was selected through stratified random sampling to participate in the wave 1 in-home interview (in 1994–1995), which was the first wave of the longitudinal study. More than 17,000 parents of respondents were also surveyed at wave 1 on their socioeconomic background, household characteristics, and perceptions of their communities. A subset of wave 1 respondents was interviewed 1 year later at wave 2, and the original wave 1 sample was contacted for re-interview at wave 3 (in 2001–2002) and again at wave 4 (in 2007–2008). In total, there were 8141 respondents with valid sampling weights who were present in all four waves of data, including the parent questionnaire (see Chen & Chantala, 2014).^1^

### Dependent variable

*Poor health* is a self-rated, time-varying measure of respondents’ overall health. At each wave of data, respondents were asked, “In general, how is your health?” Responses ranged from 1 (excellent) to 5 (poor), where higher scores indicated worse health. Prior research has found self-rated health to be a valid and consistent predictor of diagnosed illness, morbidity, and mortality (Fosse & Haas, 2009). The same measure has also been used routinely throughout the literature to examine health disparities by immigrant status, across various racial-ethnic groups, and among adolescents and young adults (Allen, McNeely, & Orme, 2016; Barnert et al., 2017; Boardman, 2006).

### Key independent variables

The key independent variables are incarceration and immigrant status. At wave 4, respondents were asked, “Have you ever spent time in a jail, prison, juvenile detention center or other correctional facility?” and, if yes, “How old were you when you (first) went to jail, prison, juvenile detention center or other correctional facility?” Using this information and respondents’ ages at each wave, we created a time-varying, wave-specific measure of whether respondents experienced their first *incarceration* before their age at each wave (1 = yes, 0 = no). Among ever-incarcerated respondents, the mean age at first incarceration was 20.85 (modal age = 18). By measuring incarceration prior to each wave, we satisfy temporal ordering by measuring poor health *after* incarceration. Also, in measuring first incarceration, we capture those confinement experiences that, theoretically, should be most consequential for health. Prior research has established that one’s first incarceration can be a life transition that sets in motion a series of accumulating disadvantages that impact well-being (Baćak et al., 2019; Sugie & Turney, 2017). According to Sugie and Turney (2017:730), “the stigma of incarceration and the stress of reentering after incarceration may be more severe for individuals experiencing their first incarceration.”

The global measure of incarceration provided by Add Health unfortunately does not offer information on the type of facility in which respondents were confined. The measure thus captures a wide range of incarceration experiences, from shorter stays in jail to lengthy stays in prison—with shorter stays in jail likely being more common (Zeng, 2019).^2^ The results should therefore be interpreted as representing average effects of incarceration on poor health across a variety of types and lengths of incarceration. The time-varying measure of incarceration that we use is consistent with prior research (Siennick & Widdowson, 2017).

*Immigrant status* was measured using the following two survey items from the wave 1 interview: “Were you born in the U.S.?” and, “Were you born a U.S. citizen?” If respondents answered “no” to both questions, they were coded as immigrants (1 = yes, 0 = no). Immigrant respondents make up just over 6% of the sample.

### Time-varying control variables

Several theoretically relevant and established correlates of incarceration and poor health were included in the analysis to minimize the threat of spuriousness. *Offending* was measured at each wave using a variety scale of ten items. These items assessed whether, in the past year, respondents self-reported that they damaged property, entered a house or building to steal something, stole something worth under $50, stole something worth over $50, sold marijuana or other drugs, took part in a physical group fight, used or threatened to use a weapon to get something from someone, pulled a knife or gun on someone, shot or stabbed someone, or hurt someone in a fight badly enough to require medical care (range = 0–10). This covariate helps to clarify whether observed health declines have associations with incarceration, independent of involvement in criminal activity (Farrington, 1995).

*Alcohol abuse* was measured at each wave and indicated the frequency with which respondents reported being “drunk or very high on alcohol” in the past year. Responses ranged from 0 (never) to 6 (every day or almost every day). Alcohol abuse is an important covariate given its links to poor health and criminal justice involvement (Kuntsche, Kuntsche, Thrul, & Gmel, 2017).

*Depressive symptoms* were measured at each wave using nine items from the CES-D that were available in each wave of the data (Radloff, 1977).^3^ At each wave, respondents were asked how often during the past 7 days they experienced the following: “you were bothered by things that don’t usually bother you,” “you could not shake off the blues, even with help from your family and your friends,” “you felt that you were just as good as other people” (reverse-coded), “you had trouble keeping your mind on what you were doing,” “you were depressed,” “you were too tired to do things,” “you enjoyed life” (reverse-coded), “you were sad,” and “you felt that people disliked you.” Responses for each item ranged from 0 (never/rarely) to 3 (most/all of the time) and were summed to create a scale where larger values reflect greater depressive symptoms (range 0–27; α = .83). Prior research has linked depressive symptoms to poor health (Sin, Kumar, Gehi, & Whooley, 2016) and incarceration (Porter & Novisky, 2017). Respondents’ *age* in years at the time of each interview was also included as a time-varying covariate.

### Time-stable control variables

A number of demographic variables were included from the wave 1 survey given their associations with poor health and incarceration in prior research. *Male* sex was measured dichotomously (1 = male, 0 = female). Race and ethnicity were measured using a set of dummy variables indicating *Hispanic*, *non-Hispanic Black, Native American*, and *Asian/other non-white*, where *non-Hispanic white* was the reference category (for each, 1 = yes, 0 = no). *English-speaking household* was based on respondents’ reports of whether English was the primary language spoken in their home (1 = yes, 0 = no). *Parental economic hardship* indicated whether the responding parent did not have enough money to pay bills (1 = yes, 0 = no). *U.S.-born parent* captured whether the respondent had a parent (mother or father) who was born in the U.S.  (1 = yes, 0 = no).

Several additional adolescent risk factors for poor health and incarceration were also included from the wave 1 survey, including neighborhood disorder, early residential mobility, and verbal intelligence. *Neighborhood disorder* was measured as the mean of parents’ responses to the following two questions: “In this neighborhood, how big a problem is litter or trash on the streets and sidewalks?” and “In this neighborhood, how big a problem are drug dealers and drug users?” Responses to each question ranged from 1 (no problem at all) to 3 (a big problem) and were summed and averaged (*r* = .47). *Early residential mobility* indicated whether respondents had moved in the past 5 years at wave 1 (0 = no, 1 = yes). Finally, *verbal intelligence* was measured using each respondent’s age-normed Add Health Picture Vocabulary Test (PVT) Score. Add Health PVT Scores come from a shorter, computerized version of the Peabody Picture Vocabulary Test (Revised) that was administered at the beginning of the wave 1 interview. There were 87 items in the Add Health PVT, and raw scores were standardized by age.

### Missing data

Item-missing data were imputed using the *mi* suite for multiple imputation with chained equations in Stata 16 (*m* = 10 imputations). Multiple imputation is a well-established approach to dealing with missing data (Royston, 2004) and the imputation model was specified using all variables in the present study (Bartlett, Frost, & Carpenter, 2011). Respondents who were originally missing information on poor health were excluded from the sample prior to analysis (von Hippel, 2007). Resulting estimates from the 10 imputed data sets were combined following Rubin’s (1987) rules. The final analytic sample consists of 7806 respondents contributing 31,202 respondent waves.^4^ Sample statistics are presented in Table 1.

**Table 1:** Descriptive statistics

	Grand Mean (SD) or %	Range
Dependent Variable		
Poor health^a^	2.14 (.91)	1–5
Key Independent Variables		
Incarceration^a^	6.70%	0–1
Immigrant	6.03%	0–1
Control variables		
Offending^a^	.64 (1.27)	0–10
Alcohol abuse^a^	1.13 (1.56)	0–6
Depressive symptoms^a^	5.96 (4.12)	0–27
Age^a^	20.30 (5.38)	11–33
Male	45.46%	0–1
Black	20.14%	0–1
Hispanic	14.92%	0–1
Native American	2.02%	0–1
Asian and other nonwhite	5.96%	0–1
English-speaking household	90.45%	0–1
Parental economic hardship	18.00%	0–1
U.S.-born parent	86.15%	0–1
Neighborhood disorder	1.49 (.53)	1–3
Early residential mobility	56.07%	0–1
Verbal intelligence	100.99 (14.33)	14–146

Descriptives are presented at the person-wave level

*N* (person waves) = 31,202

*N* (persons) = 7806

^a^Time-varying variable

### Analytic strategy

To understand the relationship between incarceration and poor health, and how it varied by immigrant status, we used hierarchical generalized linear modeling (HGLM; Raudenbush & Bryk, 2002). This approach was appropriate given that the Add Health data contain a hierarchical structure where repeated measures are nested within persons. Level 1 of the data captures time-varying (or “within-person”) effects, and level 2 captures time-stable (or “between-person”) effects. At level 1, the data contained the following time-varying independent variables, measured at each wave: first incarceration, offending, alcohol abuse, depressive symptoms, and age.^5^ At level 2, the data contained the following time-stable independent variables, measured at wave 1: immigrant status, sex, race-ethnicity, English-speaking household, U.S.-born parent, neighborhood disorder, early residential mobility, and verbal intelligence.

To leverage the panel design of Add Health, we used Allison’s (2005) between-within method, which enabled us to use respondents as their own controls. Specifically, we decomposed time-varying variables into two parts, capturing within- and between-person variation. The time-stable component was the person-specific mean of each variable, and the time-varying component was created by subtracting the person-specific mean from each observation. In our analysis, the effect of each time-varying variable on poor health was determined entirely by within-individual change, or the portion of variance that was independent from the other variables in the model (Osgood, 2010:380). This approach helped to address static and dynamic forms of selection bias, and it allowed us to examine within-individual change while controlling for sources of unobserved heterogeneity.

Our analyses proceeded in three stages. First, we used HGLM to establish the independent effects of first incarceration and immigrant status on poor health. Second, we added an interaction term to the model (*immigrant x incarceration*) to determine whether the effects of first incarceration on poor health varied by immigrant status. Third, we carried out a series of supplemental analyses to determine whether the results were robust for different groups of immigrants (Black and Hispanic immigrants; immigrants from low-middle income countries), and whether they varied by age at immigration. All models were specified to adjust for the clustered sampling design of Add Health (Chen & Chantala, 2014).

## Results

The results from HGLM analyses predicting changes to poor health are presented in Table 2. Turning first to Model 1, the results indicate that incarceration is associated with worsened health. This finding is consistent with the literature documenting that confinement contributes to health problems over time. Specifically, we found that incarceration is associated with a .021 standard deviation increase in poor health (*β* = .021, *p* < .001). This is a modest, yet notable effect size given that our models accounted for unobserved heterogeneity at the between-person level, and controlled for aging and within-person changes in offending, alcohol abuse, and depressive symptoms. Model 1 also indicates that immigrants report better health than non-immigrants, and this finding is consistent with the body of work on the immigrant paradox. Net of our other covariates, immigrant status is associated with a .042 standard deviation decrease in poor health (*β* = −.042, *p* < .001). Again this effect size is modest but nontrivial given our modeling strategy.

**Table 2:** Hierarchical generalized linear models assessing the effects of incarceration and immigrant status on poor health

Variables	Poor Health					
	Model 1			Model 2		
	*b*	(SE)	*β*	*b*	(SE)	*β*
Incarceration^a^	.122**	(.026)	.021	.124**	(.027)	.022
Immigrant	−.156**	(.034)	−.042	−.156**	(.034)	−.042
Immigrant x Incarceration	–	–		−.050	(.107)	−.002
Offending^a^	.017**	(.004)	.018	.017**	(.004)	.018
Alcohol abuse^a^	.026**	(.004)	.035	.026**	(.004)	.035
Depressive symptoms^a^	.031**	(.001)	.103	.031**	(.001)	.103
Age^a^	.016**	(.001)	.089	.016**	(.001)	.089
Male	−.165**	(.015)	−.091	−.165**	(.015)	−.091
Black	−.066**	(.019)	−.029	−.066**	(.019)	−.029
Hispanic	.108**	(.025)	.043	.108**	(.025)	.043
Native American	.152**	(.048)	.024	.152**	(.048)	.024
Asian and other nonwhite	.128**	(.033)	.034	.128**	(.033)	.034
English-speaking household	.038	(.036)	.012	.038	(.036)	.012
Parental economic hardship	.067**	(.018)	.029	.067**	(.018)	.029
U.S.-born parent	.060	(.032)	.022	.060	(.032)	.022
Neighborhood disorder	.058**	(.013)	.034	.058**	(.013)	.034
Early residential mobility	−.031*	(.014)	−.017	−.031*	(.014)	−.017
Verbal intelligence	−.003**	(.001)	−.053	−.003**	(.001)	−.053
Constant	2.001**	(.106)		2.001**	(.106)	
Variance component	.268**	(.006)		2.68**	(.006)	

Entries represent unstandardized partial regression coefficients (*b*), robust standard errors (SE), and standardized beta coefficients (*β*). Person-level means of all time-varying variables are also included in the model (not shown)

*N* (person waves) = 31,202

*N* (persons) = 7806

*p < .05; **p < .01 (two-tailed test)

^a^Time-varying variable

**Fig. 1:**
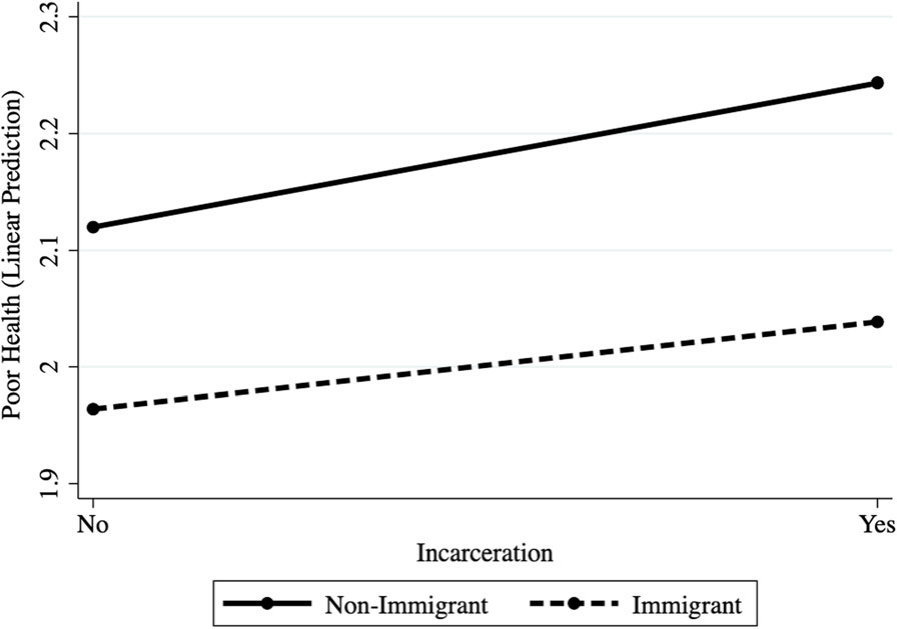
Predictive margins of the effects of incarceration on poor health, by immigrant status

An interaction term between immigrant status and incarceration is introduced in Model 2 of Table 2. The coefficient for the interaction term is nearly zero (*β* = −.002) and not statistically significant (*p* = .638), indicating that the effect of incarceration on poor health does not vary between immigrants and non-immigrants. A graph of the interaction effect is provided in Fig. 1. Overall, the findings show that, while immigrants generally have better health than native-born individuals, the associations between incarceration and health are similar between groups.

### Supplemental analyses

Several additional analyses were conducted to assess the robustness of the results. First, we determined whether the association between incarceration and poor health varied depending on whether individuals were Black or Hispanic immigrants, or whether they hailed from a low-middle income country (LMIC). Black or Hispanic immigrants may experience additional challenges in the U.S. and within correctional settings, as they are historically more likely to be subjected to mistreatment based on harmful stereotypes (Hersch, 2011). Additionally, immigrants from LMICs may be more resilient to the health damaging effects of incarceration because the conditions in their sending nations may be worse than in American correctional facilities. Individuals in LMICs are likely to grow up in stressful environments with inadequate healthcare, poor nutrition, and frequent exposure to violence (Murray et al., 2018).

To assess whether the results varied for Black and Hispanic immigrants, we created a categorical variable coded as 0 (non-immigrant; 94.0%), 1 (Black or Hispanic immigrant; 3.7%), and 2 (immigrant other race; 2.3%) and interacted it with incarceration (not shown in table form). The results showed once again that the effects of incarceration on poor health did not vary by immigrant status, regardless of whether individuals were Black or Hispanic immigrants. Specifically, interaction terms were not statistically significant and were small in magnitude (*Black or Hispanic immigrant x incarceration*: *β* = −.003, *p* = .444; *immigrant other race x incarceration*: *β* = −.004, *p* = .420).

**Fig. 2:**
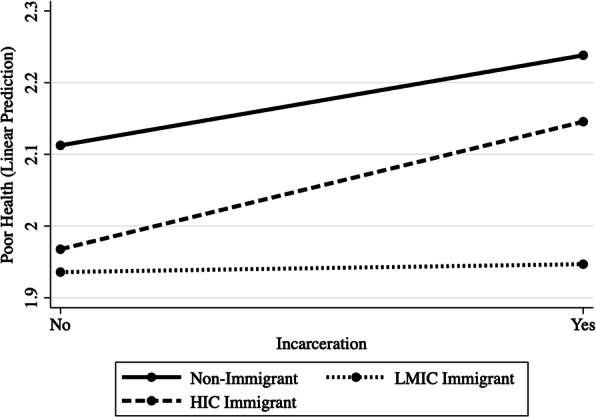
Predictive margins of the effects of incarceration on poor health, by immigrant status and country-income group. *Note:* HIC = high-income country; LMIC = low-middle income country

To determine if the effects varied depending on whether immigrants were from LMICs, we created another categorical variable using information gathered from immigrants on their country of birth, coded as 0 (non-immigrant; 94.0%), 1 (immigrant from LMIC; 4.6%), and 2 (immigrant from high-income country; 1.2%). There were over 40 different countries represented in the data, and we coded these as low-middle- or high-income according to World Bank classifications (World Bank, 2019). For example, LMICs included countries such as Cambodia, Ecuador, Haiti, Mexico, the Philippines, and Taiwan; and high-income countries (HICs) included those such as Canada, Japan, Great Britain, and Germany. There was a marginally significant interaction between *LMIC immigrant x incarceration* (*β* = −.013, *p* = .078), indicating that immigrants from LMICs were somewhat less likely than native-born individuals to exhibit health declines following incarceration. The interaction between *HIC immigrant x incarceration* was null (*β* = .004, *p* = .301), indicating no differences between immigrants from HICs and the native-born. A graph of the interaction effect can be seen in Fig. 2. These findings indicate that while immigrants from HICs may experience modest health declines following incarceration, immigrants from LMICs do not. Incarceration appeared unrelated to changes in health among immigrants from LMICs; yet the associations between incarceration and poor health were similar between HIC immigrants and the native-born.

Finally, we examined whether the findings varied by age at immigration, given that health may deteriorate with longer residence in the U.S. (Antecol & Bedard, 2006). Among foreign-born respondents, the mean age at immigration was 8.11 (modal age = 12; range 0–17). Using only immigrant respondents, we re-estimated the models with an interaction term for *age at immigration x incarceration*. Although age at immigration was inversely associated with poor health (*β* = −.088, *p* = .025), the interaction term was null (*β* = .004, *p* = .922), indicating that the effect of incarceration on health did not vary by age at immigration.

Taken together, the results show that incarceration is related to modest health declines, and that the association between incarceration and poor health is similar for immigrants and non-immigrants, for immigrants of different races, and those who immigrated at different ages. The one exception was for immigrants from LMICs, who, unlike immigrants from HICs, were somewhat less likely to experience health declines following incarceration. These results and their implications are discussed in more detail below.

## Discussion

A consistent finding in the health sciences is the immigrant paradox: where foreign-born individuals tend to fare better than native-born citizens on a host of health outcomes, despite facing significant life adversities. Yet at the same time, a wealth of research suggests that incarceration has detrimental effects on health (Massoglia, 2008). Correctional facilities are often understaffed, medical treatment services are limited, and the risks of exposure to violence and disease are high (Massoglia & Pridemore, 2015). Since incarceration is a unique life stressor—one that separates immigrants from their families and sources of capital that increase resiliency—it was unclear whether we would find support for the immigrant paradox in this context, or whether we would instead discover that immigrants and non-immigrants experience similar health declines following incarceration. Based on the results that we presented, three conclusions are warranted.

First, incarceration appears to be similarly health damaging for immigrants and non-immigrants. We found minimal support for the immigrant paradox in this particular context, and instead discovered that incarceration was associated with modest health declines for both foreign- and native-born persons. Furthermore, Black and Hispanic immigrants were just as likely to experience health declines after incarceration as immigrants of other races and ethnicities. The only exception was for immigrants who were born in low-middle income countries (LMICs). We found marginally significant differences between LMIC and HIC immigrants, where LMIC immigrants experienced no health declines following incarceration. It is possible that LMIC immigrants are somewhat more resilient to the health damaging effects of incarceration, given that the environments in their sending countries may be worse than (or similar to) the conditions within correctional facilities—especially with respect to exposure to disease, inadequate healthcare, poor nutrition, and violence. It is also possible that LMIC immigrants possess traits that allow them to better adapt to adversity (such as self-efficacy or self-determination); that they have a stronger sense of ethnic identity or greater values of familism that protect them from health declines after incarceration (Perreira et al., 2006); or, that they reside in more closely-knit communities, such as immigrant enclaves (Logan, Zhang, & Alba, 2002), that offer networks capable of bolstering social support (Feldmeyer, 2009). For other groups of foreign-born persons, however, the pattern of findings suggests that the health consequences of incarceration—although small in magnitude—are no different than for native-born persons.

Second, the findings of this study raise important questions about *how* incarceration may lead to health declines among immigrants. There are several mechanisms through which this may occur, including inhumane conditions of confinement (overcrowding, victimization, disease exposure), inadequate medical care or diagnosis within correctional facilities, limited or blocked access to medical treatment upon release, or reductions in social support and social capital. Even though we found the association between incarceration on health to be similar for immigrants and native-born individuals, it is still possible that the mechanisms underlying this effect differ across groups. For instance, the stigma of incarceration might be felt more strongly among immigrants due to the anti-immigrant sentiment that is prevalent in U.S. society. Although it is well established that immigrants commit less, not more, crime than their native-born counterparts (Light & Miller, 2018; Ousey & Kubrin, 2018), this fact is often lost on the public who routinely perceive immigrants as costly and dangerous (Flagg, 2018). Upon release, formerly incarcerated immigrants may be further ostracized by their communities, labeled as “troublesome” or “threatening,” and treated as unwanted. Formerly incarcerated immigrants can also lose social capital if their foreign-born friends and family members refuse to associate with them over fear of getting caught up in illegal activity or deported; and such losses can negatively impact health (Elgar et al., 2011). We therefore emphasize that, although we found that native- and foreign-born persons experienced similar declines in health following incarceration, the reasons why these health declines occur could differ for immigrants, and future research should examine this.

Third, our findings emphasize the importance of access to healthcare for formerly incarcerated persons, regardless of their immigrant status. Individuals released from correctional settings often face multiple barriers to healthcare (unemployment, homelessness, mental illness, addiction), including a lack of medical insurance. It is estimated that 80% of the approximately 10 million individuals released from U.S. prisons and jails each year are uninsured (Bronson & Carson, 2019; Pew Charitable Trusts, 2017; Zeng, 2019), and about 40% of incarcerated people have at least one chronic health condition, such as diabetes or hypertension (Maruschak, Berzofsky, & Unangst, 2015). Reducing health challenges among this population is important not only from a public health perspective but from a public safety one as well. Recent research shows that health problems among formerly incarcerated persons can increase crime and recidivism by reducing employment prospects and increasing financial strain (Link, Ward, & Stansfield, 2019). Accordingly, policies that seek to expand Medicaid coverage or increase access to other forms of medical insurance may be helpful for formerly incarcerated persons, including immigrants.

### Limitations

This study has several limitations that may be improved upon in future research. For one, we were unable to account for various elements of the incarceration experience that can have implications for health, such as type of institutional housing (detention, jail, or prison), the conditions of confinement that individuals were exposed to (crowding, isolation), or the length of time incarcerated. These details are important, given that they would help to clarify the link between incarceration and negative health outcomes, and would help to uncover the underlying mechanisms responsible for changes in self-rated health after correctional housing. Thus, future researchers should prioritize the use of more precise indicators of incarceration and conditions of confinement to further substantiate the link between incarceration and poor health for native and foreign-born persons. In addition, we captured only the effect of individuals’ first incarceration on health declines. Beyond the first incarceration, it is unknown precisely how many times respondents in our data were incarcerated over the study period, or at what ages they experienced each subsequent incarceration. It is possible that persons who are incarcerated repeatedly suffer worse health outcomes (Lorvick, Comfort, Kral, & Lambdin, 2018), and it remains an open question whether immigrants would be more or less resilient to the cumulative effects of repeated incarcerations. Incarceration dosage, measured as time served and the number of spells, should be considered in subsequent work (Porter & DeMarco, 2019).

It is also important to recognize that incarceration was relatively uncommon in the Add Health data, especially among immigrants. We examined some variability with respect to whether immigrants were Black or Hispanic, or if they hailed from a LMIC or a HIC, but we were unable to assess further how the health impacts of incarceration are shaped by race, ethnicity, and country of origin due to having a limited number of cases. In samples with larger incarcerated populations, this variation should be explored. We were also unable to examine if the patterns that we found varied by immigrant arrival cohorts. In the Add Health data, youth were approximately 15 years old at wave 1, and over two-thirds of immigrant youth moved to the U.S. prior to the age of 10 (between the years of 1977 and 1990). Although we did not find evidence that the effects of first incarceration on health varied by age at immigration, it is possible that different patterns would emerge in samples that included respondents who immigrated across different spans of time, or in later adolescence or early adulthood. It is possible that the patterns we found are cohort-specific, and future work should consider the effects of incarceration on the health of immigrants who entered the U.S. in more recent decades (Ro, Geronimus, Bound, Griffith, & Gee, 2015). Differences may emerge due to compositional changes across cohorts, changes in immigration policy, or changes in the global patterns of disease.

Additionally, we could not measure the characteristics of communities that individuals returned to upon release. Place matters in shaping health outcomes, and certain communities have more resources to better address residents’ healthcare needs. Subsequent research should examine the challenges immigrants and native-born individuals face in seeking health treatment services upon reentry, or if they return to communities that differ in terms of their health resources. Lastly, we measured health declines using only one item—a global indicator of poor self-rated health, which was assessed at the time of each interview. As a result, we were unable to make distinctions between physical or mental health problems or capture more temporary health declines that possibly occurred between waves of data collection. For example, even though the respondent may have been feeling well at the time of interview, and provided a positive self-assessment of health, it is possible the same person suffered health problems several months prior, which may not be reflected in the data. Research that can more precisely capture the timing of health declines and assess how incarceration leads to within-individual changes in specific types of physical and mental health problems would be useful, especially to develop tailored policies to improve the well-being of formerly incarcerated immigrant and native-born populations.

## Conclusion

In America, immigrants face a wide spectrum of adversities—from economic hardship to overt discrimination—and yet they often remain resilient anyway. However, not all adversity is created equal, and it seems that incarceration is one instance where the resilience of immigrants has run up against its limits. The broader implication is that our punishment methods ought to be reexamined if even those who are generally resilient still emerge from institutions in worse health than when they entered. Further, scholars might need to be careful not to overstate the substantive reach of the immigrant paradox. While immigrants appear strong and have a way of flourishing in the face of hardships, at least when it comes to the consequences of incarceration, they may not be wholly immune to them.

## Data Availability

Information on how to obtain the Add Health data files is available on the Add Health website (http://www.cpc.unc.edu/addhealth).

## Footnotes

1 Sampling weights were computed by Add Health investigators to address unequal probabilities of sampling and attrition across waves (Chen & Chantala, 2014). In Add Health, there is differential attrition by gender, race, and socioeconomic status. These variables, and the sampling weights, are included in the analyses. For more information on the Add Health design and data quality, see Harris (2013).

2 Data are recorded at wave 4 on the total time that individuals had been incarcerated over their lifetimes. Among ever-incarcerated respondents, the mean time served was 6 months, where 65% served a lifetime total of 1 month or less, 17% served 2–11 months, and 18% served 1 year or more. On average, immigrants were incarcerated for 3.6 months less than non-immigrants.

3 The full 20-item CES-D scale was not available in all four waves of the Add Health data. However, previous research has shown the 20-item CES-D to cluster into four subfactors—somatic-retarded activity, depressed affect, positive affect, and interpersonal relationships (Ensel, 1986)—and all four components are represented in the nine items available across all waves.

4 The inclusion of sampling weights and the requirement that respondents participated in all four waves of data resulted in a reduced sample size. Accordingly, we re-estimated our regression models without weights and without the restriction that respondents had to be in all four waves of data (*N* = 15,417 respondents). The pattern of findings was similar. The results were also consistent when using listwise deletion.

5 Age is an important covariate given that the Add Health panel design is one in which respondents were followed for more than a decade, where interviews were conducted at waves 1 (1994–1995), 2 (1996), 3 (2001–2002), and 4 (2007–2008). On average, respondents were aged 15 years at wave 1 and 28 years at wave 4.
