# Correction to: Undoing resilience: immigrant status and poor health following incarceration

**DOI:** 10.1186/s40352-021-00133-x

**Published:** 2021-02-26

**Authors:** Julie L. Kuper, Jillian J. Turanovic

**Affiliations:** College of Criminology and Criminal Justice, Florida State University, 112 S. Copeland Street, Tallahassee, FL 32306 USA

**Correction to: Health Justice 9, 5 (2021)**

**https://doi.org/10.1186/s40352-021-00129-7**

Following publication of the original article (Kuper & Turanovic, 2021), the authors noticed that one correction found in Table 2, Model 2 (“Variance Component” value, 5th column) was not implemented.

The value was printed as 2.68** but the correct value is .268**.

The original article (Kuper & Turanovic, 2021) has been updated.

## References

[CR1] Kuper JL, Turanovic JJ (2021). Undoing resilience: immigrant status and poor health following incarceration. Health Justice.

